# City of Hygeia

**Published:** 1875-12

**Authors:** 


### CITY OF HYGEIA.

Dr. Richardson gave a sketch befor the recent Social Science Congress held at Brighton, England, of a model city which he proposed to call Hygeia. He assumed the population to be 100,000, and gave them 20, 000 houses, built upon 4,000 acres of land. Tall buildings overshadowing the streets were not to be permitted; each story was limited to fifteen feet, and no house could be higher than sixty feet. They were to be built upon solid arches of brick work, and the main traffic was to be carried on in subways and underground railways. The kitchens, pantries, and wash-rooms had their location immediately under the roof, and the chimneys were to be so arranged that the smoke would be drawn into a central shaft, and after being put through a gas furnace to destroy the free carbon, would be discharged colorless into the air. Each sleeper would be allowed 1,200 cubic feet; no carpets would be displayed, but an oak flooring, to be kept clean and bright by a regular application of wax and turpentine. Hospitals were to be provided for every 5,000 people, each patient to have a well ventilated room to himself. Minute directions for eating, sleeping, dressing, and burying the dead were given, the whole scheme embodying the most approved scientific principles.

Dr. Richardson's picture is a beautiful one to contemplate, but his charming plan would not work in the long run. He suggests the possible result, from the adoption of his measures, of a reduction of the death-rate to 8 per 1,000 in the first generation, and to five or less in the next. It sounds simple enough to talk of knocking one or two per 1,000 from a death-rate, and, so long as the rate is tolerably high, such as 20 or more, the effect is not so startling, but when we come to such low figures as eight and five, the difference becomes enormous. Thus, whereas a diminution from twenty-one to twenty raises the expectation of life by only one and a half years, a fall from nine to eight raises it by nine, from six to five by twenty-one years, and from five to four by forty years. We should thus have at eight per 1,000 death-rate, an expectation of life of eighty-six years, and probable mean duration of 120, while there would be cases of old people living 160. Again, at five per 1,000 the ages would be respectively 137 years for expectation at birth, and old people living on to 250; at 4 per 1,000 the expectation would be 177, and old people would live to beyond 330. Compare these figures with Dr. Richardson's closing address, where he claims a modest ninety years as the proper length of human life. Another aspect of the case is the probable increase of population, which we may thus calculate: The mean birth-rate of England for the last thirty-five years is 33.8; if the death-rate be five the net increase would be 28.8 per 1,000. At this rate the population of Great Britain and Ireland would reach 66,000,000, by the year 1900, and by the year 2000 no less than 1,120,000,000, or about the present population of the inhabited globe. At the same time the model City of Hygeia would more than double itself by the end of the present century, while, by the end of the next, its population would be 3, 450,000, or as nearly as possible that of London to-day.

